# Perfectionism in academic settings and relationship to depression and socio-demographic characteristics

**DOI:** 10.1192/j.eurpsy.2022.1441

**Published:** 2022-09-01

**Authors:** M. Bouhamed, R. Sallemi, A. Bouaziz, I. Kallel, I. Feki, J. Masmoudi

**Affiliations:** Hospital university of HEDI CHAKER, Psychiatry A Department, Sfax, Tunisia

**Keywords:** case report, measles encephalitis

## Abstract

**Introduction:**

Acute measles encephalitis is a pathology of the central nervous system. It is most frequent in children but can also be described in adults. Given the rarity of this pathology, we present the case of this patient.

**Objectives:**

To assess perfectionism and depression and to study the relationship between these two parameters in a population of medical students.

**Methods:**

We conducted a descriptive and analytical cross-sectional study among students of the Faculty of Medicine of Sfax during the months of June, July, and August 2020. The data were collected through a self-questionnaire disseminated on the social network “Facebook”.This questionnaire included identification of socio-demographic characteristics as well as the personal history of students. Perfectionism was assessed by the Rheaume scale and depression by the Beck scale.

**Results:**

A total of 206 students participated in the survey. The mean age was 21.49 ± 1.37 years. The majority of students were female (57.2%) with a sex ratio (F/H) of 1.34. Of the participants, 39.8% were enrolled as undergraduates and 60.19% as graduate students. According to their scores on the Rheaume scale, the students were non-perfectionists (NP) in 21.4% of cases; moderately perfectionists (MP) in 70.38% of cases, and highly perfectionists (HP) in 8.25% of cases. Severe depression was objectified in 7.3% of the cases. Students living alone were less perfectionist (p=0.01). Perfectionism score was higher in depressed students (35 ± 6.34) versus (31.428 ± 6.37) with a statistically significant correlation ( p ≤ 0.001).

**Conclusions:**

It is true that perfectionism is an essential element for academic success. However, screening and prevention of depression are deemed necessary given its significant association with perfectionism.

**Disclosure:**

No significant relationships.

